# Degenerative Cervical Myelopathy: How to Identify the Best Responders to Surgery?

**DOI:** 10.3390/jcm9030759

**Published:** 2020-03-11

**Authors:** Rocco Severino, Aria Nouri, Enrico Tessitore

**Affiliations:** 1 Division of Neurosurgery, IRCCS Neuromed, 86077 Pozzilli (IS), Italy; severinorocco@gmail.com; 2 Department of Neurosurgery, Hôpitaux Universitaires de Genève (HUG), 1205 Geneva, Switzerland; enrico.tessitore@hcuge.ch

**Keywords:** degenerative cervical myelopathy (DCM), surgical outcome, MRI, DTI, FA, ADC, signal changes spinal canal, neurophysiology, SSEP, MEP

## Abstract

Surgery is the only definitive treatment for degenerative cervical myelopathy (DCM), however, the degree of neurological recovery is often unpredictable. Here, we assess the utility of a multidimensional diagnostic approach, consisting of clinical, neurophysiological, and radiological parameters, to identify patients likely to benefit most from surgery. Thirty-six consecutive patients were prospectively analyzed using the modified Japanese Orthopedic Association (mJOA) score, MEPs/SSEPs and advance and conventional MRI parameters, at baseline, and 3- and 12-month postoperatively. Patients were subdivided into “normal” and “best” responders (<50%, ≥50% improvement in mJOA), and correlation between Diffusion Tensor Imaging (DTI) parameters, mJOA, and MEP/SSEP latencies were examined. Twenty patients were “best” responders and 16 were “normal responders”, but there were no statistical differences in age, T2 hyperintensity, and midsagittal diameter between them. There was a significant inverse correlation between the MEPs central conduction time and mJOA in the preoperative period (*p* = 0.0004), and a positive correlation between fractional anisotropy (FA) and mJOA during all the phases of the study, and statistically significant at 1-year (r = 0.66, *p* = 0.0005). FA was significantly higher amongst “best responders” compared to “normal responders” preoperatively and at 1-year (*p* = 0.02 and *p* = 0.009). A preoperative FA > 0.55 was predictor of a better postoperative outcome. Overall, these results support the concept of a multidisciplinary approach in the assessment and management of DCM.

## 1. Introduction

Degenerative cervical myelopathy (DCM) is typically a chronic condition, commonly involving patients older than 55 years [1], and represents the most common cause of spinal cord injury in the industrialized world [2]. The progressive reduction of spinal canal diameter due to degeneration of the cervical spine, including the vertebrae, posterior longitudinal ligament, ligamentum flavum, intervertebral disk [3], results in a compression of the spinal cord, arterial perfusion to the nervous tissue, and consequent spinal cord ischemia [4,5]. DCM can be a highly disabling condition causing motor and sensory dysfunction that ultimately result in a reduced quality of life.

The diagnosis of DCM is based on clinical examination, and subsequently confirmed using imaging, and sometimes neurophysiological techniques such as sensory (SSEPs) and motor evoked potentials (MEPs). Studies using conventional MRI have shown that specific characteristics can correlate with neurological status and surgical outcome. The most commonly studied parameters include T1-weighted hypointensity or T2-weighted hyperintensity signals of the spinal cord and the number of compressed levels. It is believed that signal changes represent a wide-ranging set of pathological sequelae. Edema and gliosis are thought to result in demyelination and Wallerian degeneration, and are typically associated with T2 hyperintensity signal changes in the absence of T1 hypointensity [6]. After prolonged compression or significant dynamic injury, myelomalacia and loss of grey matter occurs [7,8,9,10], typically reflected by T1 hypointensity signal changes. However, T2 hyperintensity presents in 58%–85% of DCM patients, but it is present in 2.3% of people in the general population as well, making it a sensitive measure for diagnosis, but limiting it in terms of predicting surgical outcome. Contrarily, T1 hypointensity has been found to be a good predictor of suboptimal surgical outcome but its low prevalence in DCM of about 20% of patients limits its clinical utility [11].

In recent years, a newer MRI technique, the diffusion tensor imaging (DTI), has demonstrated an ability to identify the degenerative changes of the compressed spinal cord even in the early phases of DCM. [12,13] In the neural tissue, DTI imaging estimates the directionality and the diffusivity of water molecules through tissues and nervous fibers through two values: The fractional anisotropy (FA) and the apparent diffusion coefficient (ADC). These scalar parameters are inversely proportional and, in a damaged and demyelinated spinal cord water molecules diffuse in all different directions resulting in lower FA and higher ADC studies values [10,14], while in the normal population FA has higher scores because of intact myelin sheaths. [14,15] Moreover, several studies have shown a correlation between preoperative FA, ADC values, and clinical condition of DCM patients [13,16]. 

Neurophysiological studies are also useful diagnostic tools in the detection of functional alterations in nerve conduction in DCM. Several studies have demonstrated significant alterations in both SSEPs and MEPs in DCM patients [17,18,19]; specifically, a predictive value for the postsurgical outcome has been shown for median nerve SSEPs [19,20,21]. 

In a previous study, we were able to demonstrate the importance of combining clinical, radiological, and neurophysiological data in the assessment of DCM patients, in order to better identify those with optimal surgical outcomes [22]. The aim of the present study is to evaluate the correlation between DTI, neurophysiological parameters and neurological status, in both the preoperative and postoperative periods, in order to define a new multidisciplinary diagnostic approach that could identify the best candidates for decompressive surgery. 

## 2. Methods

We performed a prospective analysis of clinical, radiological, and neurophysiological data of thirty-six consecutive patients (13 males, 23 females; mean age: 57.05 years) suffering from DCM and operated between June 2012 and June 2018. Inclusion criteria are detailed in Table 1. All subjects gave their written informed consent for inclusion before they participated in the study. Research ethics board approval was obtained by the Institutional Ethical Board (NAC n. 11-194). A complete clinical, radiological, and neurophysiological evaluation of DCM, consisting of cervical MRI with DTI sequences, the modified Japanese Orthopedic Association (mJOA) score by Keller et al. [23], and neurophysiological assessments (both MEPs and SSEPs), was performed for all patients in the preoperative period and repeated at 3- and 12-months after surgery. The choice of the surgical approach depended on the predominant side of compression and on the surgeon’s preferences. The posterior approach was chosen in patients with a multilevel compression and with preservation of the cervical lordosis on plain X-rays. Fusion was added in cases with radiological instability according to White and Panjabi criteria [24].

The mJOA score was used to measure the severity of DCM and segregated into patients with normal function (mJOA = 16–17), grade 1 (mJOA = 12–15), and grade 2 (mJOA = 8–11) myelopathy. We calculated the improvement of DCM using the mJOA recovery rate proposed by Hirabayashi, as follows: [(Postoperative mJOA-preoperative mJOA)/(17-preoperative mJOA) × 100] [25]. “Best responders” were identified by improvement of 50% or more in the postoperative period [26]. Patient improving below 50% or remaining stable (at least no deterioration) were defined as “normal responders”. 

### 2.1. Radiological Assessment

All patients underwent a cervical 3 Tesla MR with the following sequences: Sagittal fast spin echo T2 (FSET2) TE 102 ms, TR 3500 ms, slice thickness 3 mm, axial gradient echo T2 TE 14 ms, TR 676 ms, slice thickness 3 mm, sagittal FSE T1, TE 10 ms, TR 900 ms, slice thickness 3 mm, sagittal diffusion tensor imaging (DTI) TE 58 ms, TR 4000 ms, *b*-value 700, slice thickness 2 mm, 25 directions with calculation of the apparent diffusion coefficient (ADC) and fractional anisotropy (FA). 

**Figure 1:**
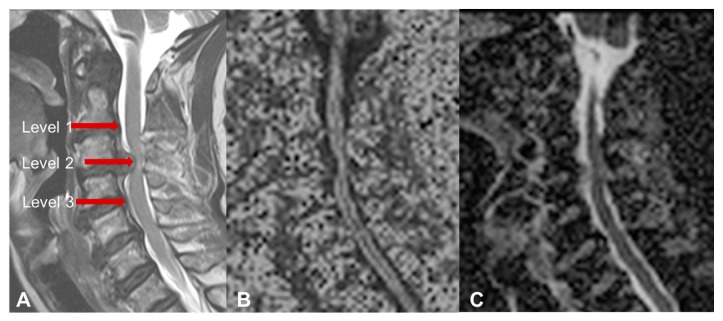
**A**: Measurement levels for fractional anisotropy (FA) and the apparent diffusion coefficient (ADC): The surgical level, or the narrowest point of the cervical stenosis in patients with multilevel compression (level 2), and the intervertebral levels above and below it (level 1 and 3). **B, C**: Diffusion tensor imaging (DTI) sequences for FA and ADC measurement, respectively.

The images were obtained in the preoperative period and at 3- and 12-months after surgery. The FA and the ADC were measured with isometric ROIs at three levels: The surgical level, or the narrowest point of the cervical stenosis in case of multilevel compression (level 2), and the nearest noncompressed intervertebral levels above and below (level 1 and 3, respectively—Figure 1). The presence and the extension of T2 hyperintensity, and the presence of T1 hypointensity was also investigated. 

The midsagittal diameter of the spinal canal at the site of greatest compression, was calculated on sagittal T2 images in both preoperative and postoperative controls (at three months) and used this to examine the expansion rate of the spinal canal after the surgery, using the formula: [(Postoperative–Preoperative AP diameter)/Preoperative AP diameter] × 100.

### 2.2. Electrophysiological Assessment

All patients had undergone electrophysiological evaluation with SSEPs and MEPs. The Quadriceps Combined Test [27] was used for the analysis of the MEPs. Furthermore, calculation of the preoperative and postoperative (3-months and 1-year) central conduction time (TCC), corrected for the age and size, and the amplitude ratio of the MEP was obtained in all patients. 

For SSEPs, the peak latencies of responses were recorded at Erb’s point (N9), the C2 spinous process (N13), and the scalp (N20) for the median nerve. For the tibial nerve, we calculated the latencies N8–N22 (popliteal fossa to L1) and N22 (L1 spinous process). Unfortunately, several patients did not perform a complete postoperative SSEP analysis according to our criteria as a result of a mismatch between different neurophysiological diagnostic units. Therefore, only the SSEP analysis on the preoperative data was conducted. 

### 2.3. Statistical Analysis

Data were analyzed using an unpaired t-test to compare the mJOA, midsagittal diameter of the spinal canal, DTI parameters, and MEP/SSEP values, during all the phases of the study, in the “best responders” and “normal responders” to surgery. A Pearson correlation analysis was then performed to assess the correlation between pre and postoperative FA and ADC values, mJOA score, and MEP/SSEP values. Fischer exact tests were performed to determine a specific preoperative FA threshold that could be predictive of a better postoperative outcome. Fisher exact tests were also used to investigate if general “risk factors” (T2 hyperintensity, Tobacco use, diabetes, and clinical history >6 months) were related to worse outcome. A *p*-value < 0.05 was considered to be statistically significant. 

## 3. Results

### 3.1. Clinical Demographics and Outcome

Twenty-six patients (72.2%) were operated by an anterior approach, including an anterior discectomy and fusion (ACDF) in 20 patients and an anterior corpectomy and fusion in six patients. Ten patients (27.3%) were treated with posterior decompression by laminectomy with or without fusion. Based on the mJOA score, there were 5/36 patients with normal function, 25/36 patients with grade 1 myelopathy, and 6/36 patients with grade 2 myelopathy. There were no patients with a preoperative grade 3 myelopathy.

**Figure 2:**
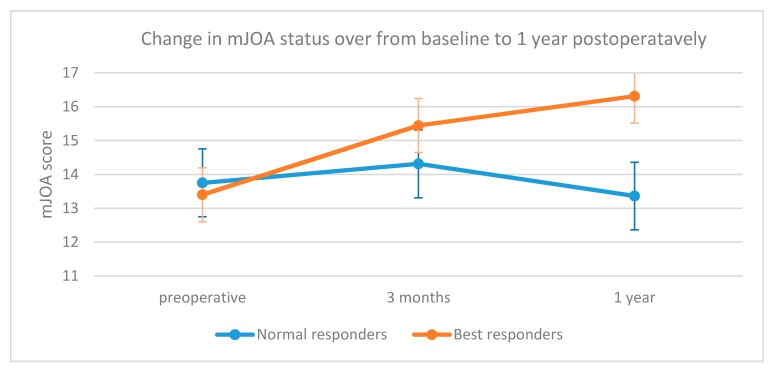
Difference of trends of the average modified Japanese Orthopedic Association (mJOA) scores in the “best responders” and “normal responders” patients. The improvement from the preoperative score to the 1-year value in the “best responders” group was significant (*p* = 0.001), as the difference between the 1-year values of the “best responders” (mean = 16.3) and the “normal responders” (mean = 13.3) patients (*p* = 0.001).

The preoperative mJOA average value was 13.5, while the postoperative mean values were 14.9 at 3-months and 15.1 at 1-year, respectively. According to the Hirabayashi recovery ratio, 20 patients (55.5%) were considered “best responders”; the difference between the mJOA improvement of the “best responders” and “normal responders” patients at 1-year was statistically significant (*p* = 0.001, Figure 2). The difference between the mean age of the “best responders” and “normal responders” was not statistically significant (58.9 ± 13.2 vs. 54.6 ± 13.1, *p* = 0.34).

There was no statistical difference between the two groups concerning both age (58.9 ± 13.2 vs. 54.6 ± 13.1, p: 0.34) and the investigated “risk factors” (T2 hyperintensity, p: 0.13; smoke, p: 0.22; diabetes, p: 0.83; clinical history > 6 months, p: 0.14. Table 2).

### 3.2. Radiological Results

The mean midsagittal canal diameter was 5.12 ± 1.4 and 8.92 ± 2 mm in the preoperative and postoperative period, respectively, with a mean expansion rate of 97.4%. Considering our cases as “best responder” and “normal responder” patients, we found no significant differences between the average values of both postoperative midsagittal diameters (8.98 ± 2.3 vs. 8.84 ± 1.6 mm, *p* = 0.8) and expansion rates (100.1% vs. 93.9%, *p* = 0.8).

**Figure 3:**
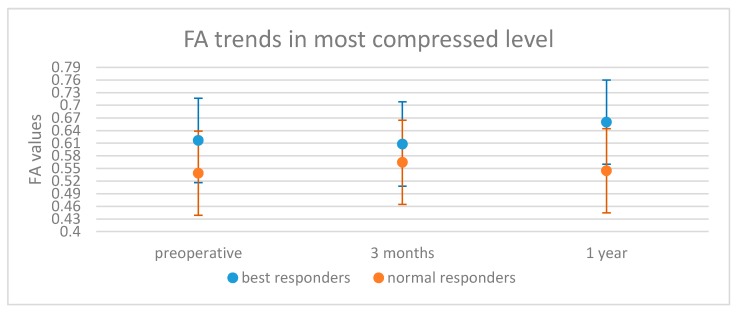
Differences between fractional anisotropy average values of the most compressed level in best responders (blue) and normal responders (red) patients.

Concerning the preoperative DTI parameters, the preoperative FA values were significantly higher in the “best responders” than the “normal responders” (0.63 ± 0.06 vs. 0.57 ± 0.08, *p* = 0.03). Six patients were excluded from the postoperative analysis because they presented with artefacts on their MRIs related to implanted metallic devices. In the remaining 30 patients, the average FA value remained higher in the “best responders” group at both 3-months (0.62 ± 0.08 vs. 0.58 ± 0.09) and statistically significantly different at 1-year (0.68 ± 0.07 vs. 0.55 ± 0.11, *p* = 0.004, Table 2). Furthermore, FA at the most stenotic level was significantly lower in the “normal responder” group preoperatively and at 1-year (*p* = 0.02 and *p* = 0.009, respectively—Figure 3, Table 2).

The “best responders” group had a preoperative FA > 0.55 in 71.5% of patients compared with only 28.5% of the “normal responders”. This difference was statistically significant (p = 0.014), and suggests that a preoperative FA > 0.55 can be considered as a predictor of a better postoperative outcome.

T2 hyperintensity in the preoperative MRI was found in 12/16 (75%) of the “normal responders” patients and in 10/20 (50%) in the “best responders” group; however, this difference was not significantly different (*p* = 0.13). The preoperative average FA was similar in patients with and without T2 hyperintensity (0.595 vs. 0.596, *p* = 0.9). No T1 hypointensity was evident in any of our patients.

Concerning the ADC, the average value between the “normal responder” group compared to the “best responders” group was higher in the preoperative period (1.54 vs. 1.40) and at the 3-month follow-up (1.40 vs. 1.27), but lower at the 1-year control (1.37 vs. 1.40 in the “best responders” group). These results did not show a statistically significant difference, and we did not consider a lower ADC as a predictive factor for good recovery.

### 3.3. Neurophysiological Results

**Figure 4:**
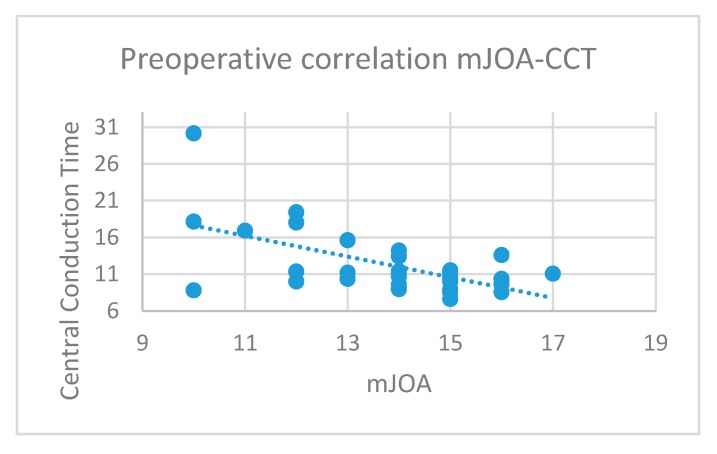
Preoperative abnormal values of motor evoked potentials (MEPs) were related to worse mJOA scores: This inverse correlation was statistically significant (r = −0.59, *p* = 0.0004).

Concerning the MEPs, a significant inverse correlation between the CCT and mJOA values in the preoperative period (*p* = 0.0004, R = −0.59, Figure 4) was found.

**Figure 5:**
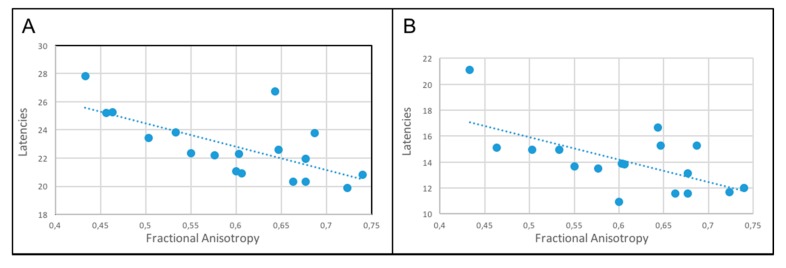
**A:** Inverse correlation between preoperative FA values and L1 spinous process (N22) (*p* = 0.001). **B**: Inverse correlation between preoperative FA values and popliteal fossa to L1 (N8–N22) (*p* = 0.007).

We found no correlation between abnormal preoperative SSEPs and preoperative mJOA scores. Regarding the relationship between SSEP and FA values, we observed a significant inverse correlation between preoperative FA and N22, N8–N22 latencies (*p* = 0.001 and *p* = 0.007, respectively (Figure 5), there was no statistically significant correlation with the other SSEPs otherwise. The same correlation was found postoperatively but due to the mismatch of the test conducted in different diagnostic centers we were not able to conduct any statistical analysis.

### 3.4. Correlation between FA, mJOA Values, and Neurophysiological Parameters

**Figure 6:**
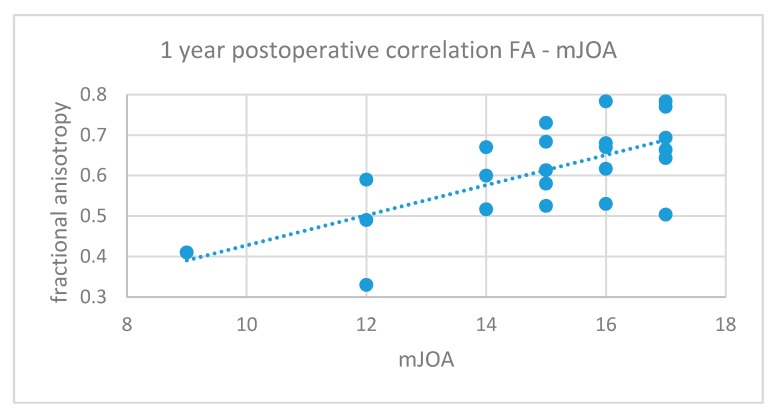
Positive correlation between preoperative FA and mJOA at 1 year (*p* = 0.004, r = 0.66).

A positive correlation between FA values and corresponding mJOA scores during all the phases of the study was found. However, this correlation was significant only for the 1-year postoperative values (*p* = 0.0005, R = 0.66, Figure 6).

**Figure 7:**
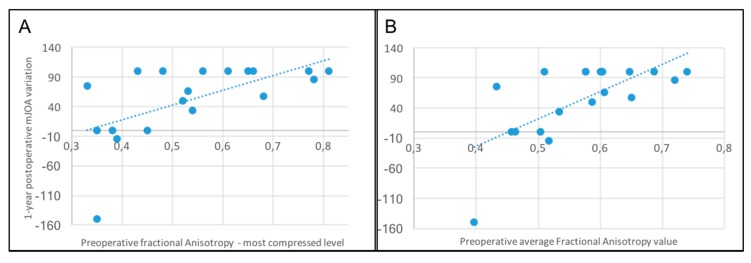
**A:** Significant correlation between preoperative FA value at the most compressed level and the 1-year postoperative variation of the mJOA (*p* = 0.002). B: Significant correlation between preoperative FA average value and the 1-year postoperative variation of the mJOA (*p* = 0.0002).

Moreover, a direct correlation was found between higher preoperative FA values, and the postoperative variation of the mJOA at 1 year. This result was significant considering both the FA at the most compressed level and the average value of all the three considered levels (*p* = 0.002, r = 0.66 and *p* = 0.0002, r = 0.75, respectively—Figure 7).

Concerning the relationship between FA and MEP values, an inverse correlation was found between TCC values and FA scores in the preoperative period, but this result was not statistically significant (r = −0.33; *p* = 0.08).

## 4. Discussion

DCM is a complex and potentially disabling condition. The time for surgical intervention is usually dictated by the degree of neurologically severity, with moderate to severely impaired patients recommended for surgery, whereas mildly impaired patients may be offered surgery or careful observation. [28] Although most patients with DCM will improve after surgery, it remains challenging to accurately predict good responders. These difficulties are due, in part, to the different pathophysiological causes of chronic cervical myelopathy such as ischemic degeneration of the neural tissue due to hypoperfusion, loss of motoneurons in the anterior horns [29], membrane damages, and conduction decline [30]. Presently, we are faced with a true *“paradigm shift”*, passing from an era where the goal of surgery for DCM was to stop the disease progression, to an era where surgery seems to be able to improve patients’ status in most of the cases, having thus a favorable impact on DCM patients’ quality of life [31].

Unfortunately, there is a lack of a standard diagnostic protocol that can provide prognostic information for DCM patients. Past notions about the usefulness of MRI findings in DCM, such as the presence of T2 hyperintensity, have shown to be largely nonspecific with regards to the severity of myelopathy in specific patients and the capacity of postoperative neurological improvement [8,13,32,33,34]. Having said this, T2 hyperintensity seems to have some potential utility when measured in terms of sagittal extent, or when skip lesions are observed [11].

It remains unclear if age impacts outcome, while some have demonstrated that age is a predictive variable, others have found no such relationship for the postsurgical recovery rate [35,36]. In our study we found that T2 hyperintensity, Tobbaco use, diabetes, and a clinical history >6 months, are not related to a worse outcome. Furthermore, multilevel compression was also not predictive of a poorer neurological outcome. While, these results are consistent with some studies found in literature [8,37,38,39,40], other studies, notably those derived from the AOSpine multicenter studies on DCM, have shown contradictory results [11,41]. Our results may partially be due to our relatively small cohort and may have been underpowered to detect statistically significant differences.

In contrast with the results reported by various authors [41,42,43,44], we found that surgical outcome was independent from both the age and the spinal canal diameter before surgery. In fact, we observed no statistical difference between the spinal canal midsagittal diameter of “best responders” and “normal responders”, both in the preoperative and postoperative periods. This may be partially due to dynamic injury mechanisms that contribute to DCM and that are not necessarily influenced by canal diameter.

New MRI techniques, such as the DTI, in combination with neurophysiological assessment can help identify those patients with higher probabilities of improving after surgical decompression [22]. Similarly to previous research [45], FA in “best responders” were significantly higher than those of the “normal responders”, both preoperatively and 1-year follow-up. Moreover, there was also a statistical difference in the FA values of the most stenotic level in all the phases of the study between the two groups, and a significant relationship between a preoperative FA > 0.55 at the most compressed level and a better clinical outcome (RR > 50%) at 1-year. This is consistent with the concept that FA can be used in the assessment of the degree of severity of DCM with greater accuracy. [12,13,14,34,43] Our results support what has been previously suggested—higher values of preoperative FA can be considered as a positive prognostic factor of functional recovery [13,34,37]. It was interesting to note that, FA values were not different between best and normal responders at 3-months, and this potentially indicates a 3-month FA MRI may be too early to detect changes.

Neurophysiological parameters have been reported as useful diagnostic tools for DCM. It has been previously shown that MEPs have a good diagnostic value in the setting of DCM, perhaps even more so than SSEPs [19]. Our study shows support to this concept in that preoperative abnormal CCT was related to a worse clinical condition and a lower mJOA (*p* = 0.0004). The significant correlation between preoperative FA and N22, N8–N22 latencies in the preoperative period are challenging to interpret—further research needs to be done in this area to better understand this relationship and how it may be useful in diagnosis and outcome prediction, or if this was a spurious finding.

Several authors stated that median SSEPs can be used for both diagnostic and prognostic purposes in DCM patients. Lyu et al. [20] stated that normal median SSEPs are related to a better prognosis; Morishita et al. [21] showed that an early improvement of the N18 was related to a good outcome at 3-months after surgery. Restuccia et al. [19] found that the SSEP improvement was related to a better clinical outcome, especially in those patients with an isolated loss of N13. These results confirm that SSEPs are a useful tool for the diagnosis of a cervical myelopathy. Unfortunately, in our study we were not able to perform a complete SSEP analysis in all our patients in the postoperative period. Nevertheless, we observed in the preoperative data, a correlation between fractional anisotropy and neurophysiological parameters and in particular tibial SSEPs, suggesting the existence of connection a between a radiological information and a clinical data.

### Limitations

The greatest limitation of the present study is the relatively small cohort and the retrospective nature of our analysis. However, few studies have assessed the combination of electrophysiology in conjunction with the commonly used clinical and advanced MRI parameters. While our study did not support some of the findings of previous authors that remain supported with low evidence, our findings with regards to FA are in accordance with others [34,45,46] and strongly support the effectiveness of DTI analysis in both assessing the clinical status and predicting the surgical outcome in DCM patients.

Unfortunately, the use of different neurophysiological methods in the postoperative period did not allow us to assess the effectiveness of changes in these parameters in the postoperative setting.

Lastly, patients with preoperative mJOA scores of 17 were included; however, while these patients did not show clear neurological impairment as assessed by the mJOA, they exhibited neurophysiological evidence and/or objective clinical signs such as hyper-reflexia, Hoffman’s sign.

## 5. Conclusions

Our results validate the concept that the current “ordinary” assessment of DCM should be upgraded with new diagnostic techniques. DTI could be considered not only a complementary diagnostic analysis, but rather a crucial tool in order to identify the best candidates to surgery. Neurophysiological parameters, in particular MEPs, correlate with the clinical condition of the patient, and could therefore be considered as an additional diagnostic tool in the preoperative period. The inclusion of DTI sequences in the preoperative study provides also prognostic information, enhancing the presurgical evaluation of DCM patients. Our findings suggest that FA values are most useful preoperatively and at 1-year follow-up, and may not be useful at 3-months postoperatively. The inclusion of electrophysiology and DTI measurements may enhance the diagnostic process and may be effective at augmenting the predictive capacity of previously described prediction models.

## Figures and Tables

**Table 1:** Inclusion and exclusion criteria.

Inclusion Criteria	Exclusion Criteria
Age: 30–81 years	Contraindication to perform MR
Cervical stenosis at MR	Epileptic patients
Need for decompressive surgery	Pregnant
Clinical signs of myelopathy	Cancer or infection
Complete follow up at three months and one year	Previous cervical spine surgery

**Table 2:** Differences in characteristics between best responders and normal responders.

Characteristics		Best Responders	Normal Responders	*p*-Value
		*n* = 20	*n*=16	
Age		58.9 ± 13.2	54.6 ± 13.1	0.34
Smoke		0.30	0.50	0.22
T2 hyperintensity		0.50	0.75	0.13
Diabetes		0.05	14.2%	0.83
Symptoms > 6 month		81.8%	0.75	0.14
Midsagittal diameter (mm)	Preoperative	5.10 ± 1.4	5.15±1.4	0.44
	Postoperative	8.98 ± 2.3	8.84±1.6	0.48
Cord expansion rate		100.1%	93.9%.	0.8
	Preoperative	0.63 ± 0.06	0.57±0.08	0.03 *
Average FA	Three months	0.62 ± 0.08	0.58±0.09	0.3
	One year	0.68 ± 0.07	0.55±0.11	0.004 *
	Preoperative	0.63 ± 0.15	0.55±0.11	0.02 *
Surgical level FA	Three months	0.62 ± 0.09	0.56±0.14	0.33
	One year	0.67 ± 0.08	0.54±0.09	0.009 *

*: Statistically signifant result.
